# 20-Hydroxyecdysone Protects against Oxidative Stress-Induced Neuronal Injury by Scavenging Free Radicals and Modulating NF-κB and JNK Pathways

**DOI:** 10.1371/journal.pone.0050764

**Published:** 2012-12-11

**Authors:** Jun Hu, Chun Xia Luo, Wei Hua Chu, You An Shan, Zhong-Ming Qian, Gang Zhu, Yan Bing Yu, Hua Feng

**Affiliations:** 1 Department of Neurosurgery, Southwest Hospital, Third Military Medical University, Chongqing, People's Republic of China; 2 Department of Neurosurgery, China-Japan Friendship Hospital, 2, Beijing, People's Republic of China; Albany Medical College, United States of America

## Abstract

Oxidative stress plays an important role in the pathological processes of ischemic brain damage. Many antioxidants have been shown to protect against cerebral ischemia injury by inhibiting oxidative stress both in vitro and in vivo. 20-Hydroxyecdysone (20E), an ecdysteroid hormone, exhibits antioxidative effects. For the work described in this paper, we used an in vitro oxidative damage model and an in vivo ischemic model of middle cerebral artery occlusion (MCAO) to investigate the neuroprotective effects of 20E and the mechanisms related to these effects. Treatment of cells with H_2_O_2_ led to neuronal injury, intracellular ROS/RNS generation, mitochondrial membrane potential dissipation, cellular antioxidant potential descent, an increase in malondialdehyde (MDA) and an elevation of intracellular [Ca^2+^], all of which were markedly attenuated by 20E. Inhibition of the activation of the ASK1-MKK4/7-JNK stress signaling pathway and cleaved caspase-3 induced by oxidative stress were involved in the neuroprotection afforded by 20E. In addition, 20E reduced the expression of iNOS protein by inhibition of NF-κB activation. The neuroprotective effect of 20E was also confirmed in vivo. 20E significantly decreased infarct volume and the neurological deficit score, restored antioxidant potential and inhibited the increase in MDA and TUNEL-positive and cleaved caspase-3-positive cells in the cerebral cortex in MCAO rats. Together, these results support that 20E protects against cerebral ischemia injury by inhibiting ROS/RNS production and modulating oxidative stress-induced signal transduction pathways.

## Introduction

Ischemic brain injury is a leading cause of death and disability in the aged population in many countries. It is characterised by the disruption of cerebral blood flow and lack of oxygen to the affected area. Brain damage following cerebral ischemia develops from a complex series of pathophysiological events that evolve in time and space. The processes of excitotoxicity, peri-infarct depolarisation, inflammation, and apoptosis within the ischemic penumbra are proposed [1]. During these processes, large amounts of free radicals (reactive oxygen and nitrogen species) were produced [2], [3]. Excessive production of free radicals (reactive oxygen and nitrogen species) causes an imbalance between pro-oxidants and antioxidants and damages biomolecules, such as proteins, lipids, and nucleic acids. Such phenomena are collectively called oxidative stress [4]. Cumulative evidence suggests that oxidative stress is a fundamental mechanism of ischemic brain injury [2], [3], [5], [6]. Reactive oxygen species (ROS) such as superoxide ions and hydroxyl radicals have been considered important mediators causing oxidative damage after cerebral ischemia [7]. ROS generation after cerebral ischemia damage membranes (lipolysis) and mitochondria and induce an increase in intracellular calcium concentration ([Ca^2+^]i) [8]. Besides, ROS initiate apoptosis signaling pathways [9]. For example, ROS are potent inducers of c-Jun N-terminal kinase (JNK), which is an important subgroup of the mitogen-activated protein kinase (MAPK) superfamily [10]. Activation of JNK has been observed in many neuronal oxidative damage models and appears to be critical in mediating neuronal cell apoptosis [11]. The upstream kinase apoptosis signal-regulating kinase 1(ASK1) is a specific target for ROS. ROS readily activate ASK1, which further leads to the activation of JNK via MKK4 and MKK7 [10], [12]. Reactive nitrogen species (RNS), such as nitric oxide (NO), are also important mediators that cause oxidative damage after cerebral ischemia [13], [14]. The expression of inducible nitric oxide synthase (iNOS) is upregulated under cerebral ischemic conditions via the activation of nuclear transcription factor κB (NF-κB) [15], and this phenomenon is accompanied by increased generation of NO. Interaction between NO and superoxide generates the strong oxidant peroxynitrite, which causes neuronal cell injury. Because ROS/RNS play an important role in cerebral ischemia injury, identifying neuroprotective agents that target the regulation of intracellular ROS/RNS levels and signaling pathways initiated by ROS/RNS has become an important strategy in the development of novel neuroprotective therapies for cerebral ischemia.

20-hydroxyecdysone (20E) is a polyhydroxylated steroid invertebrate hormone found in insects and few plants [16]. It regulates the molting, metamorphosis and reproduction of arthropods [16], [17]. A substantial body of evidence suggests that 20E may have significantly positive pharmacological properties in mammals, such as stimulating protein synthesis [18], [19], promoting carbohydrate and lipid metabolism [20], inhibiting apoptosis [21], [22], inducing stem cell differentiation, and so on. This is consistent with the use of several 20E-containing plant species in Chinese herbs, such as Achyranthes bidentata Blume and Cyanotis arachnoidea C. B. Clarke. Recently, 20E has been shown to have significant antioxidant activity by radical scavenging tests in vitro and in vivo. Lipid peroxidation in microsomal and mitochondrial fractions of rat liver is inhibited by 20E to a similar degree as by vitamin D3 (cholecalciferol), a known ROS scavenger [23], [24], [25]. The antioxidative activity of 20E is as high as that of known inhibitors of lipid peroxidation, such as diethyl para-phenylenediamine and thylenediamine tetraacetate [26], [27]. Moreover, we have previously shown that 20E can alleviate neurological deficits induced by experimental subarachnoid hemorrhage in rabbits [28], [29] and protect PC12 cells from hypoxia injuries [30]. These studies suggest that 20E may have a potent neuroprotective effect by inhibition of oxidative stress. Therefore, the present study was designed to investigate the neuroprotective effects of 20E in B35 neuroblastoma cells with hydrogen peroxide (H_2_O_2_) insult and to uncover the mechanisms behind 20E's neuroprotective action. In addition, we observed the neuroprotective effects of 20E in rats with ischemia following middle cerebral artery occlusion (MCAO). Our results demonstrate that 20E protected against oxidative stress-induced neuronal injury in vitro and brain ischemic insult in vivo. Therefore, we suggest that 20E could be considered a potential protective agent for the treatment of brain ischemic insult.

## Materials and Methods

### Materials

20-Hydroxyecdysone (20E) was purchased from Tauto Biotech Co., Ltd (Shanghai, China). Fetal bovine serum and Dulbecco's modified Eagle's medium (DMEM) were purchased from Gibco (Carlsbad, CA, USA). Hydrogen peroxide (H_2_O_2_), propidium iodide (PI), N-([3-(aminomethyl)phenyl]methyl) ethanimidamide dihydrochloride (1400W), pyrrolidine dithiocarbamate (PDTC), 3-(4,5-dimethylthiazol-2-yl)-2,5-diphenyltetrazolium bromide (MTT), and 2,3,5-triphenyltetrazolium chloride (TTC) were purchased from Sigma Chemical Co. (St. Louis, MO, USA). Dichlorofluorescin diacetate (DCF-DA), 5,5′,6,6′-tetrachloro-1, 1′,3,3′-tetraethylbenzimidazol carbocyanine iodide (JC-1) and Fluo-3 AM were purchased from Molecular Probes (Eugene, OR, USA). The in situ cell death detection kit was purchased from Roche Diagnostics (Indianapolis, IN, USA). The reagent kits for determining lactate dehydrogenase (LDH) and malondialdehyde (MDA) were purchased from Nanjing Jiancheng Institute of Biological Engineering (Nanjing, China). The reagent kits for determining total antioxidant capability (T-AOC), superoxide dismutase (SOD) activity, glutathione peroxidase (GPX) activity level and level of nitric oxide (NO) in the culture media, bicinchoninic acid kit for protein determination (BCA kit), anti-mouse IgG horseradish peroxidase (HRP) and anti-rabbit IgG-HRP were purchased from the Beyotime Institute of Biotechnology (Haimen, Jiangsu, China). Rabbit monoclonal antibodies against cleaved caspase-3 and rabbit polyclonal antibodies against p-NF-κB (p65 subunit), p-ASK1, MKK4, p-MKK4, MKK7 and p-MKK7 were purchased from Cell Signal Technology, Inc. (Beverly, MA, USA). Rabbit polyclonal antibodies against ASK1 and iNOS and mouse monoclonal antibodies against p-JNK, JNK and p- IκBα were purchased from Santa Cruz Biotechnology (Santa Cruz, CA, USA).

### Cell culture

B35 rat neuroblastoma cells (ATCC, Manassas, VA) were cultured in DMEM containing 10% fetal bovine serum at 37°C in a water-saturated atmosphere with 5% CO_2_. The culture medium was replenished at 3- to 4-day intervals based on the doubling time of B35 cells. Cells were kept in the same medium and treated with varying concentrations of H_2_O_2_ for the required time. Cells received 20E pretreatment for 24 h before H_2_O_2_ exposure.

### Assessment of cell viability and damage

An MTT assay was used to estimate cell viability. MTT assays are based on the conversion of MTT to formazan crystals via mitochondrial dehydrogenases in viable cells. In brief, B35 cells were exposed to different concentrations of H_2_O_2_ alone for 12 h or different concentrations of 20E alone for 24 h or were pretreated with different concentrations of 20E before the onset of H_2_O_2_ treatment. After these treatments, MTT was added to the culture medium at a final concentration of 0.5 mg/mL and incubated at 37°C for 4 h. Then the medium was discarded, and DMSO was added to solubilize the formazan reaction product with shaking for 5 min. The optical density (OD) was spectrophotometrically measured at 490 nm using a microplate reader (Bio Tek Instruments, Winooski, VT) with DMSO as the blank. Cell viability of the control group not exposed to either H_2_O_2_ or 20E was defined as 100%. Other groups' cell viabilities were expressed as a percentage of the control.

It has been previously established that lactate dehydrogenase (LDH) release correlates linearly with the number of damaged cells after toxic insult [31]. To confirm cell injury, LDH activity in the medium and in cells was determined according to the protocols of an LDH kit. To summarize, the cells were treated as described for the MTT assay, and then the supernatant was collected for LDH measurement in the cell-free medium. The cell pellet and the cells remaining on the multiwell were lysed in 0.5 mL of lysis buffer (0.5% Triton X-100 in 0.1 M potassium phosphate buffer, pH 7.0) for LDH measurement in the cells. The release of intracellular LDH to the extracellular medium was measured by determining the enzyme activity and was expressed as a percentage of total cellular activity. Colorimetric absorbance was measured at 450 nm with a microplate reader.

### Assessment of cell apoptosis

#### TUNEL assay

A TUNEL assay was used to evaluate cell apoptosis. TUNEL is a common method for detecting the DNA fragmentation that results from cell apoptosis. For the in vitro experiment, the cells were treated as described for measurement of LDH release. Then the cells were fixed in 4.0% paraformaldehyde for 20 min at room temperature and washed three times in PBS buffer. Fixed cells were subjected to a TUNEL assay via an in situ cell death detection kit according to the manufacturer's specifications. Finally, these cells were stained with PI dye at room temperature for 10 min. The stained cells were washed three times with PBS. The results were visualized using confocal laser microscopy (Leica TCS). A standard fluorescein filter set was used to visualize the green fluorescence of fluorescein at 530 nm and the red fluorescence of PI at 620 nm. The cells with green fluorescence and the cells with red fluorescence were counted to quantify the apoptotic process. Data are expressed as the ratio of TUNEL-positive cells to total cells. In the in vivo experiments, the rats were treated as described for the ischemic model and drug administration. The TUNEL assay was performed to reveal apoptosis-induced DNA fragmentation in brain paraffin sections of experimental rats. In brief, the sections were deparaffinized with xylene, hydrated in serially-diluted ethanol, and then treated with protease K (20 µg/mL) for 15 min at room temperature. Reaction buffer containing terminal deoxynucleotidyl transferase was directly applied to tissue sections and incubated for 1 h at 37°C. After being washed with PBS, the sections were incubated with anti-digoxigenin peroxidase conjugate for 30 min at room temperature and subsequently developed color in peroxidase substrate. The nuclei were counterstained with hematoxylin. A TUNEL-positive cell was indicated by brown coloration. The total cell number and the number of TUNEL-positive cells were determined in five areas of the cerebral cortex in every section using a square micrometer eyepiece at 200× magnification. Five sections were selected from every experimental rat. Data are expressed as the ratio of TUNEL-positive cells to total cells.

### Immunocytochemistry

To confirm the results of the TUNEL assay, immunocytochemistry stain for cleaved caspase-3 was used. The cells were treated as described for the TUNEL assay. The cells were fixed with 4% paraformaldehyde for 20 min at room temperature and washed three times in PBS buffer. Fixed cells were blocked by incubation in PBS with 5% normal goat serum and 0.5% triton X-100 at room temperature for 30 min. Then the cells were incubated with anti-cleaved caspase-3 antibody overnight at 4°C. After cells were washed three times with PBS, they were incubated with red fluorescent secondary antibody (cy3) for 1 h at 37°C. Finally, these cells were stained with DAPI dye at room temperature for 10 min. The stained cells were washed three times with PBS. The results were visualized using confocal laser microscopy (Leica TCS). A standard fluorescein filter set was used to visualize the blue fluorescence of DAPI at 488 nm and the red fluorescence of cy3 at 570 nm. The cells with blue fluorescence and the cells with red fluorescence were counted to quantify the apoptotic process. Data are expressed as the ratio of cleaved caspase-3-positive cells to total cells.

### Measurement of intracellular ROS

The fluorescent probe DCF-DA was used to estimate intracellular ROS accumulation. Cell-permeable DCF-DA is rapidly oxidized to highly fluorescent DCF in the presence of ROS. The DCF fluorescence intensity thus correlates linearly with the amount of intracellular ROS formed. The cells were plated in 6-well plates and glass bottom cell culture dishes and pretreated with 50 or 400 µM 20E for 24 h. Then these cells were treated with 150 µM H_2_O_2_ for 12 h. The treated cells were loaded with 30 µM DCF-DA. After the cells were incubated at 37°C for 30 min, the dye was removed, and the cells were washed three times with PBS. Finally, the results were visualized using confocal laser microscopy (Leica TCS SP5). Alternatively, fluorescence intensity was monitored using flow cytometry (BD Immunocytometry Systems).

### Assessment of mitochondrial membrane potential

A cationic dye, JC-1, was used to monitor the mitochondrial membrane potential. At low mitochondrial membrane potentials, JC-1 continues to exist as a monomer and produces a green fluorescence (emission at 527 nm). At high mitochondrial membrane potentials, JC-1 forms J aggregates (emission at 590 nm) and produces a red fluorescence. JC-1 exhibits potential-dependent accumulation in mitochondria indicated by a fluorescence emission shift from green to red. Thus, mitochondrial depolarization is indicated by a decrease in the red/green fluorescence intensity ratio. The cells were treated as described for measurement of intracellular ROS, and then JC-1 (10 µM) was loaded. After 30 min incubation at 37°C, the dye was removed, and the cells were washed three times with PBS. Finally, the results were visualized using confocal laser microscopy (Leica TCS SP5). Alternatively, fluorescence intensity was monitored using flow cytometry (BD Immunocytometry Systems).

### Measurement of intracellular free calcium concentration

The fluorescent Ca^2+^ indicator Fura-3/AM was used to monitor the intracellular free calcium concentration ([Ca^2+^]i). The cells were plated in glass bottom cell culture dishes and then were loaded with the Fluo-3/AM (5 µM) for 40 min at 37°C in Hank's buffer. After loading, cells were washed three times with Hank's buffer to remove excess extracellular Fura-3/AM. Fluorescence measurements were performed using confocal laser microscopy (Leica TCS SP5). Fluo-3 was excited at 488 nm, and fluorescence was measured at wavelengths of 515 nm every 5 s. After the baseline [Ca^2+^]i was observed for 60 s, 1.5 mM H_2_O_2_ was added to the dishes, and changes in [Ca^2+^]i were measured. Cells were pretreated with 50 or 400 µM 20E for 1 h. All images (about 120 images) from the scanning were processed to analyze changes of [Ca^2+^]i in a single cell. The change in intracellular calcium was expressed as relative fluorescence intensity (RFI).

### Determination of total antioxidant capability (T-AOC), superoxide dismutase (SOD) activity, glutathione peroxidase (GPX) activity level and malondialdehyde (MDA) level

To protect against oxidative stress, cells usually possess defense mechanisms, including their own intracellular antioxidants (such as antioxidant enzymes and cellular metabolites) [4]. Therefore, in this study, we investigated T-AOC, SOD activity, GPX activity level and MDA level in vitro and in vivo. The T-AOC, SOD activity, GPX activity level and MDA level were measured with the respective commercial kits. In brief, the cells were harvested following treatment by a cell scraper. After three washes with cold PBS, cells were lysed in ice-cold buffer (50 mmol/L Tris-HCl, 5 mmol/L EDTA, and 1 mmol/L DTT) by sonication. Then, lysed cells were centrifuged at 14000×g for 5 min at 4°C to remove debris. The supernatant was subjected to the measurement of T-AOC GPX activity level, SOD activity and MDA level in vitro experiments. At the same, cortical tissues were collected following treatment and placed into an ice-cold RIPA lysis buffer. The samples were then homogenized by a Polytron homogenizer followed by centrifugation at 14000×g for 5 min at 4°C. The supernatant was collected for T-AOC, GPX activity level, SOD activity and MDA level measurement in vivo experiments. Measurement of T-AOC was based on the Ferric Reducing Ability of Plasma (FRAP) method. Ferric ions were reduced by antioxidants to ferrous ions, and the optical density was measured at 590 nm with a microplate reader. Intracellular SOD activity was determined using a xanthine and xanthine oxidase system to produce superoxide. The superoxide oxidizes nitroblue tetrazolium to form a blue formazan salt, and the optical density at 560 nm was measured with a microplate reader. Determination of GPX activity level was based on measurement of the coupled oxidation of NADPH during glutathione reductase recycling of oxidized glutathione from GPX-mediated reduction of t-butyl peroxide. The optical density was measured at 340 nm with a microplate reader. MDA levels were measured by a method based on a reaction with thiobarbituric acid. The optical density at 532 nm was measured with a microplate reader.

### Measurement of nitric oxide (NO) in the culture media

To estimate cellular NO stress, the amount of NO in cellular culture supernatants was determined by measuring the accumulated level of nitrite in the supernatant using a nitrite detection kit. In brief, supernatants were collected after cell treatment. Then 50 µL of cell medium or NaNO_2_ standard samples was mixed with 100 µL of Griess reagent at room temperature. The optical density at 540 nm was measured with a microplate reader. The standard curve produced by NaNO_2_ was generated for quantification.

### Preparation of cellular protein extracts

The cells were harvested following treatment. After three washes with cold PBS, cells were lysed directly on culture plates with RIPA lysis buffer (50 mmol/L Tris–HCl, 150 mmol/L NaCl, 1% Nonidet-40, 0.5% sodium deoxycholate, 1 mmol/L EDTA, 1 mmol/L PMSF) with protease inhibitors (pepstatin 1 mg/mL, aprotinin 1 mg/mL, leupeptin 1 mg/mL) for 30 min on ice. The lysate was centrifuged at 14000×g for 15 min at 4°C. The supernatant consisting of total cellular protein extracts was collected and stored at 70°C until use. The nuclear and cytoplasmic proteins were extracted according to the instructions of the nuclear and cytoplasmic protein extraction kit (Beyotime Institute of Biotechnology, Haimen, Jiangsu, China). In summary, cells were washed in ice-cold PBS and then were gently scraped off the bottom of the flask into the media using a cell scraper. The media were centrifuged for 5 min at 1200 rpm at 4°C, and the pellet was dissolved with cytoplasmic protein extraction agent A supplemented with PMSF. After vortexing for 5 s and incubation on ice for 15 min, the cytoplasmic protein extraction agent B was add into the tubes, which were then vortexed for 5 s and incubated on ice for 5 s. The samples were centrifuged for 5 min at 14000×g at 4°C, and the supernatant, consisting of the cytosolic fraction, was immediately stored at 70°C until use. The pellet was resuspended in nuclear protein extraction agent supplemented with PMSF. After vortexing the tubes 15–20 times for 30 min and centrifuging for 10 min at 14000×g, the supernatants containing nuclear extracts were obtained. These protein extracts were quantified using a BCA Protein Assay kit (BPA, Beyotime Institute of Biotechnology, Haimen, Jiangsu, China). The nuclear and cytoplasmic proteins were subjected to immunoblotting to determine the activity of NF-κB in cells.

### Western blot analysis

Equal amounts of protein were fractionated by sodium dodecyl sulfate–polyacrylamide gel electrophoresis and subsequently transferred to a nitrocellulose membrane (Amersham Pharmacia Biotech, UK). The membrane was incubated in fresh blocking buffer (5% nonfat milk in Tris-buffered saline containing 0.1% Tween 20) at room temperature for 1 h and then incubated with primary antibodies overnight at 4°C. The primary antibodies used in this study were anti-iNOS (1∶500), anti-phosphorylated NF-κB (p65 subunit) (1∶500), anti-phosphorylated IκBα (1∶500), anti-ASK1(1∶500), anti-phosphorylated ASK1(1∶1000), anti-MKK4 (1∶1000), anti-phosphorylated MKK4(1∶1000), anti-MKK7(1∶1000), anti-phosphorylated MKK7(1∶1000), anti-JNK(1∶500), and anti-phosphorylated JNK(1∶500). The blots were washed three times in TBS-T for 5 min and then incubated with specific peroxidase-coupled secondary antibodies (anti-mouse IgGHRP or anti-rabbit IgG-HRP) at room temperature. The bound antibodies were visualized using an enhanced chemiluminescent detection system (Amersham Pharmacia Biotech, Piscataway, NJ, USA) and then exposed to X-Ray films (Kodak X-Omat, Rochester, NY, USA). The images were scanned with a GS800 Densitometer Scanner (Bio-Rad, Hercules, CA), and OD data were analyzed using Quantity One software. In these analyses, β-actin and Lamin B1 were used as an internal reference.

### Ischemic model and drug administration

Adult male Sprague-Dawley (SD) rats weighing 250–300 g (Third Military Medical University, Chongqing, China) were housed in an environmentally controlled room at 22±2°C, with a relative humidity of 55±5%, a 12-h light/ = dark cycle, and food and water ad libitum. All experimental procedures were performed in accordance with the Third Military Medical University Guide for the Care and Use of Laboratory Animals. All surgery was performed under sodium pentobarbital anesthesia, and all efforts were made to minimize suffering. Focal cerebral ischemia was produced by a modification of the intraluminal occlusion as described by Feng Zhang et al [32]. In brief, rats were anesthetized with pelltobarbitalum natricum (40 mg/kg, intraperitoneal injection). The right common carotid artery (CCA), external carotid artery (ECA) and internal carotid artery (ICA) were isolated. The ICA was occluded temporarily with a small clip at the peripheral site of the bifurcation. Then a small hole was cut in the CCA, and a 6-0 nylon monofilament with a tip that was blunted (0.2–0.22 mm) with a coagulator was inserted. After the clip at the ICA was removed, the nylon was then inserted at least 18–20 mm from the carotid bifurcation to occlude the middle cerebral artery. After 2 h of MCAO, the suture was slowly withdrawn to allow for 24-h reperfusion. The sham-operated rats received all surgical procedures, but the suture was not advanced beyond the internal carotid bifurcation. Throughout the procedure, rectal temperature was maintained at 37±0.5°C with a temperature-regulated heating pad. The right femoral artery was cannulated, and blood pressure and heart rate were measured throughout the study by a multi-channel physiological monitor (RM6280c, China). Changes in regional cerebral blood flow (rCBF) before, during, and after MCAO were evaluated in the rat by a DRT-4 laser Doppler fluxmeter (Moor Instruments Ltd., Devon, UK). The 20E-treated group was administered 20E (20 and 40 mg/kg, intraperitoneal injection) at the onset of reperfusion, whereas the sham-operated group and operated group were given the same amount of distilled water.

### Neurological deficits

At 24 h after reperfusion, the neurological behavioral assessment was performed by an observer who was blinded to the experimental conditions in the surviving animals. Neurological deficits were scored on a 0–4 scale: 0 = no apparent deficits; 1 = contralateral forelimb flexion; 2 = decreased grip of contralateral forelimb while the tail was pulled; 3 = spontaneous movement in all directions, contralateral circling if pulled by the tail; 4 = spontaneous contralateral circling [33].

### Measurement of infarct volume

Animals were anesthetized with pelltobarbitalum natricum and sacrificed by decapitation 24 h after reperfusion. Brains were quickly removed, and five 2-mm consecutive coronal slices were made beginning from the anterior pole. The sections were stained with 2% TTC in saline for 30 min at 37°C and photographed. Then photographs of the five sections were analyzed for infarct size by a blinded observer using image analysis software (Image-Pro Plus 6.0). Data are expressed as percentage infarct volume. Percentage infarct volume was calculated as described: [(V C–V L)/V C]×100%, V C is the volume of the control hemisphere, and V L is the volume of the non-infarcted tissue in the lesioned hemisphere [34].

### Immunohistochemistry

At 24 h after MCAO reperfusion, rats were anesthetized with pelltobarbitalum natricum and then perfused transcardially with 200 mL of saline followed by 300 mL of 4% paraformaldehyde solution. Brains were removed and fixed in 4% paraformaldehyde solution at 4°C for three days and then dehydrated and embedded in paraffin blocks. Five-micrometer-thick sections were cut through the dorsal hippocampus. Immunohistochemistry was performed with antibodies for cleaved caspase-3. In brief, tissue sections were deparaffinized and wetted with graded alcohol. The tissue sections were incubated with 3% H_2_O_2_, 5% normal goat serum and 0.5% triton X-100 at room temperature for 30 min each. After anti-cleaved caspase-3 antibody was applied to the sections and incubated overnight at 4°C, the sections were incubated with anti-mouse IgG horseradish peroxidase for 1 h at 37°C. The sections were subsequently incubated with diaminobenzidine (DAB) and counter-stained with hematoxylin. A cleaved caspase-3-positive cell was indicated by brown coloration. The total cell number and the number of cleaved caspase-3-positive cells were determined in the five areas of the cerebral cortex in every section using a square micrometer eyepiece at 200× magnification. Five sections were selected from each experimental rat. Data are expressed as the ratio of cleaved caspase-3-positive cells to total cells.

### Statistical analysis

Data were expressed as the mean ± S.E.M., and statistical significance was assessed by one-way analysis of variance (ANOVA) and Tukey's test. Neurological behavior scores were analyzed by using a nonparametric Kruskal–Wallis test and Dunn's Multiple Comparison Test. *p*<0.05 was considered to be significant.

## Results

### 20E attenuated oxidative stress-induced injury in B35 neural cells

**Figure 1:**
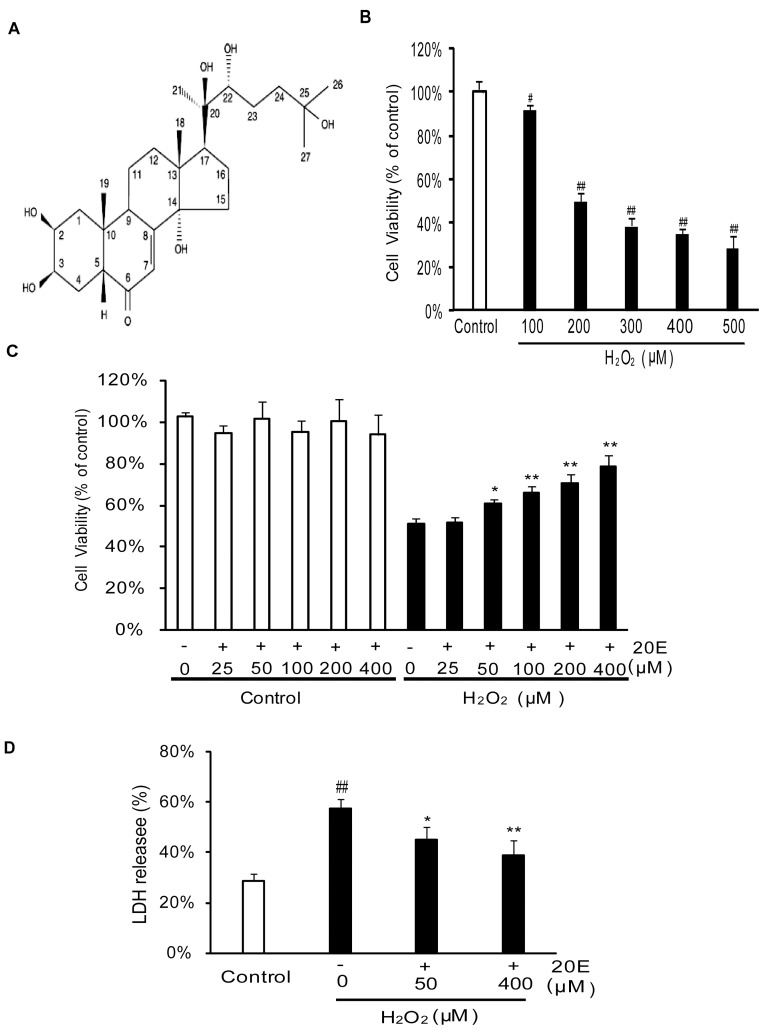
Protective effects of 20E against H_2_O_2_-induced cytotoxicity in B35 cells. A: Chemical structure of 20E. B: Concentration-dependent effect of H_2_O_2_ on cell viability in B35 neural cells. B35 neural cells were exposed to different concentrations of H_2_O_2_, and then cell viability was assessed 12 h later by an MTT assay. C: Inhibition of H_2_O_2_-induced decrease in cell viability by 20E in B35 neural cells. Cells were exposed to 20E at the concentrations of 25 to 400 µM for 24 h or were pretreated with 20E (25 to 400 µM) for 24 h prior to a 12 h incubation with H_2_O_2_. Cell viability was assessed, as measured by an MTT assay. D: Cell injury was assessed by testing LDH release. Cells were pretreated with 20E (50 or 400 µM) for 24 h before a 12 h incubation with H_2_O_2_, and then LDH release (% of total) was calculated as the percentage of LDH in the medium versus total LDH activity in the cells. Data are reported as the means ± S.D. from three independent experiments. Non-treated cells served as controls. ^#^ *p*<0.05 vs. Control, ^##^ *p*<0.01 vs. Control, ^*^ *p*<0.05 vs. H_2_O_2_ alone, ^**^ *p*<0.01 vs. H_2_O_2_ alone.

The MTT assay revealed that H_2_O_2_ decreased cell viability in a concentration- dependent manner. As shown in Fig. 1B, the viability of the cells exposed to H_2_O_2_ at concentrations of 100 to 500 µM for 12 h decreased to 91.81±2.45%, 49.87%±4.39%, 38.86±3.18%, 35.28±2.22% and 28.60±5.74% of the control value, respectively. Based on this result, a treatment of 200 µM H_2_O_2_ for 12 h was used to induce B35 neural cell injury in subsequent experiments. To examine the neuroprotective effects of 20E on H_2_O_2_-induced cytotoxicity, B35 neural cells were pretreated with 20E for 24 h before the onset of H_2_O_2_ treatment. Application of 20E at 25 µM did not attenuate H_2_O_2_-induced cytotoxicity. We therefore increased the amount of 20-Hydroxyecdysone to 50, 100, 200, or 400 µM. The cell viability significantly increased to 60.65±2.15% (*p*<0.05 in comparison to the H_2_O_2_ treatment group), 65.75%±3.53% (*p*<0.01 in comparison to the H_2_O_2_ treatment group), 70.56±4.18% (*p*<0.01 in comparison to the H_2_O_2_ treatment group), and 78.40±5.42% (*p*<0.01 in comparison to the H_2_O_2_ treatment group) of the control value at 50, 100, 200 and 400 µM, respectively (Fig. 1c). In another experiment, 48 h of treatment with various concentrations (25–400 µM) of 20E alone did not lead to any apparent increase in the viability of cells (Fig. 1c). These findings demonstrate that 20E protects B35 neural cells against H_2_O_2_-induced cytotoxicity in a dose-dependent manner.

**Figure 2:**
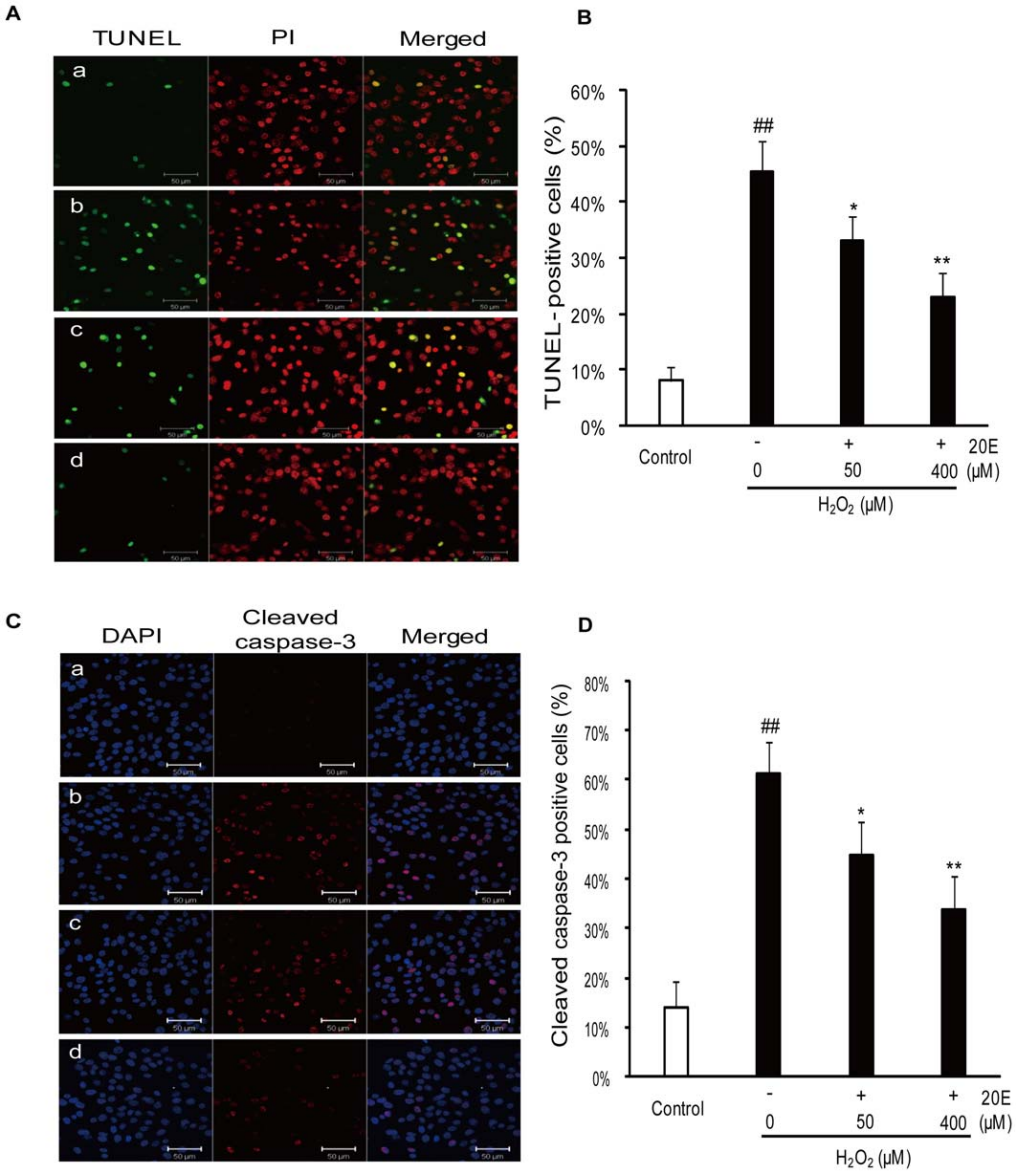
Protective effects of 20E against H_2_O_2_-induced apoptosis in B35 cells. Cells were pretreated with 50 or 400 µM of 20E for 24 h before a 12 h incubation with H_2_O_2_. a: control; b: H_2_O_2_ treatment; c: 50 µM 20E-pretreatment for 24 h before H_2_O_2_ exposure; d: 400 µM 20E-pretreatment for 24 h before H_2_O_2_ exposure. A: apoptosis was examined by TUNEL analysis with TUNEL staining (green) and propidium iodide (PI, red) counterstaining. Scale bar, 50 µm. B: Histogram showing the percentage of TUNEL-positive cells in the total cell population after different treatments. C: apoptosis was examined by immunocytochemistry for cleaved caspase-3 with cleaved caspase-3 (red) and dihydrochloride (DAPI, blue) counterstaining. Scale bar, 50 µm. D: Histogram showing the percentage of cleaved caspase-3-positive cells in the total cell population after different treatments. Data are reported as the means ± S.D. from three independent experiments. Non-treated cells served as controls. ^##^ *p*<0.01 vs. Control, ^*^ *p*<0.05 vs. H_2_O_2_ alone, ^**^ *p*<0.01 vs. H_2_O_2_ alone.

The neuroprotective effects of 20E were confirmed by the LDH assay, TUNEL analysis and immunocytochemistry stain (cleaved caspase-3). As shown in Fig. 1D, the cells treated with 200 µM H_2_O_2_ for 12 h caused an increase in LDH release (% of total), from 28.91±2.52% to 57.37±4.03%. However, pretreatment with 50 or 200 µM 20E for 24 h before H_2_O_2_ exposure decreased the LDH release to 45.20±4.82% (*p*<0.05 in comparison to the H_2_O_2_ treatment group) and 39.11±5.95% (*p*<0.01 in comparison to the H_2_O_2_ treatment group), respectively. Similarly, the TUNEL analysis showed that H_2_O_2_ stimulation increased the percentage of TUNEL-positive cells from 8.17±2.32% to 45.30±5.78%, which was significantly reduced to 32.99±4.51% (*p*<0.05 in comparison to the H_2_O_2_ treatment group) and 22.97±4.40% (*p*<0.01 in comparison to the H_2_O_2_ treatment group), respectively, by pretreatment with 50 or 400 µM 20E (Fig. 2A, B). Consistent with the TUNEL analysis data, the percentage of cleaved caspase-3-positive cells increased from 14.00±5.15% to 61.27±6.52% after a challenge with H_2_O_2_. However, the percentage of cleaved caspase-3-positive cells was significantly reduced to 44.67±6.93% (*p*<0.05 in comparison to the H_2_O_2_ treatment group) and 33.93±6.34% (*p*<0.01 in comparison to the H_2_O_2_ treatment group), respectively, when the cells were pretreated with 50 or 400 µM 20E for 24 h before H_2_O_2_ exposure (Fig. 2C, D).

### 20E reverses H_2_O_2_-induced intracellular ROS accumulation and cellular antioxidant potential descent

**Figure 3:**
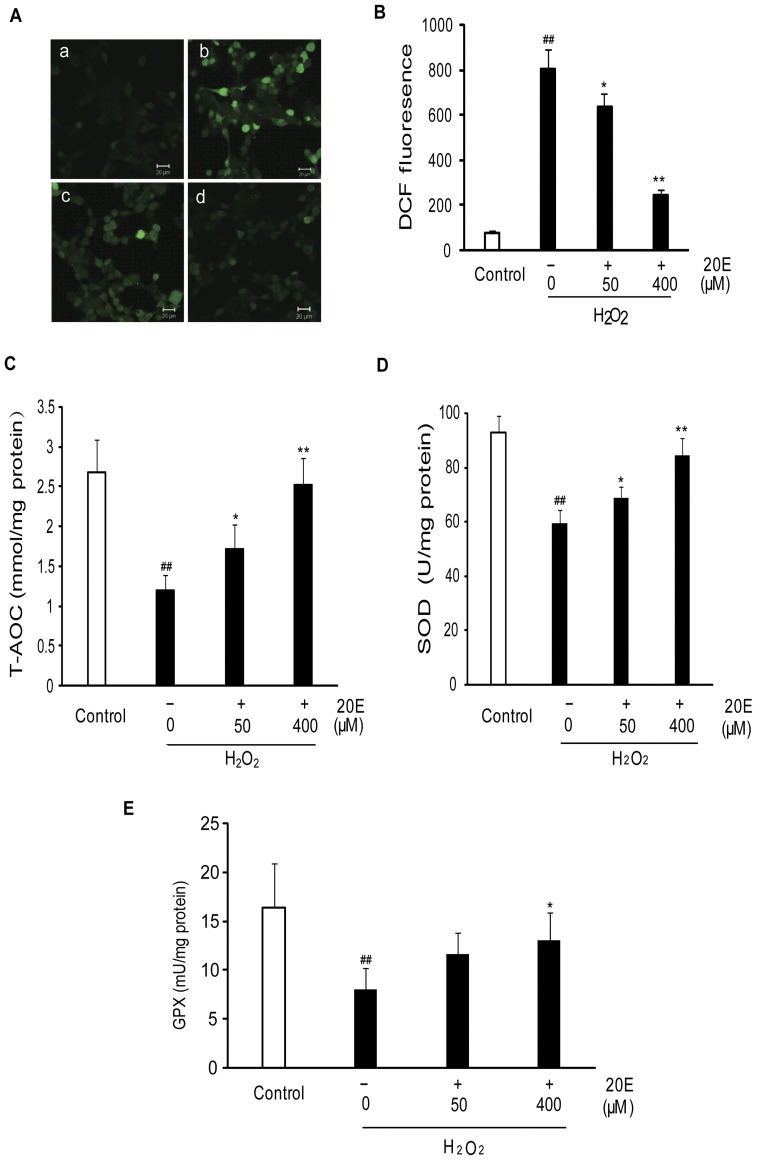
Effect of 20E on H_2_O_2_-induced oxidative stress in B35 cells. Cells were pretreated with 50 or 400 µM 20E for 24 h before a 12 h incubation with H_2_O_2_. Then these treated cells were loaded with 30 µM DCF-DA for 30 min. A: Intracellular ROS levels were determined based on DCF fluorescence by confocal laser microscopy. a: control; b: H_2_O_2_ treatment; c: 50 µM 20E-pretreatment for 24 h before H_2_O_2_ exposure; d: 400 µM 20E-pretreatment for 24 h before H_2_O_2_ exposure. B: DCF fluorescence was measured by flow cytometry. Histogram showing the intracellular fluorescence intensity of DCF in different treatment cells. Data are reported as the means ± S.D. from three independent experiments. Non-treated cells served as controls. ^##^ *p*<0.01 vs. Control, ^*^ *p*<0.05 vs. H_2_O_2_ alone, ^**^ *p*<0.01 vs. H_2_O_2_ alone. C: Histogram showing cellular T-AOC in different treatment cells. D: Histogram showing cellular SOD activity in different treatment cells. E: Histogram showing cellular GPX levels in different treatment cells. Data are reported as the means ± S.D. from four independent experiments in triplicate. Non-treated cells served as controls. ^##^ *p*<0.01 vs. Control, ^*^ *p*<0.05 vs. H_2_O_2_ alone, ^**^ *p*<0.01 vs. H_2_O_2_ alone.

Oxidative stress is the result of an imbalance between the generation of ROS and the compensatory response from the endogenous antioxidant network [4]. To examine whether 20E mediates its neuroprotective effect by inhibiting oxidative stress, we investigated the effect of 20E on intracellular ROS and cellular antioxidant potential after H_2_O_2_ exposure. In this study, a fluorescent probe, DCF-DA, was used as a specific marker for quantitative intracellular ROS formation. T-AOC, SOD activity and GPX activity level were used to evaluate cellular antioxidant potential. After B35 neural cells were exposed to 200 µM H_2_O_2_ for 12 h, the intracellular DCF-fluorescence intensity significantly increased (*p*<0.01 in comparison to the control group) (Fig. 3 A, B), and the T-AOC, SOD activity and GPX activity level were reduced from 2.67±0.41 to 1.19±0.2 mmol/mg protein, 92.83±6.1 to 59.42±4.8 U/mg protein and 16.4±4.56 to 8.07±2.13 mU/mg protein, respectively (Fig. 3 C–E). However, pretreatment with either 50 or 400 µM 20E for 24 h before H_2_O_2_ exposure dramatically decreased the intracellular DCF-fluorescence intensity (Fig. 3 A, B) and increased the T-AOC, SOD activity and GPX activity level to 1.72±0.3 or 2.52±0.34 mmol/mg protein, 68.65±4.34 or 84.36±6.52 U/mg protein and 11.64±2.23 or 13.09±2.84 mU/mg protein, respectively (Fig. 3 C–E).

### 20E attenuates oxidative stress-induced mitochondrial membrane potential dissipation and lipid peroxidation

**Figure 4:**
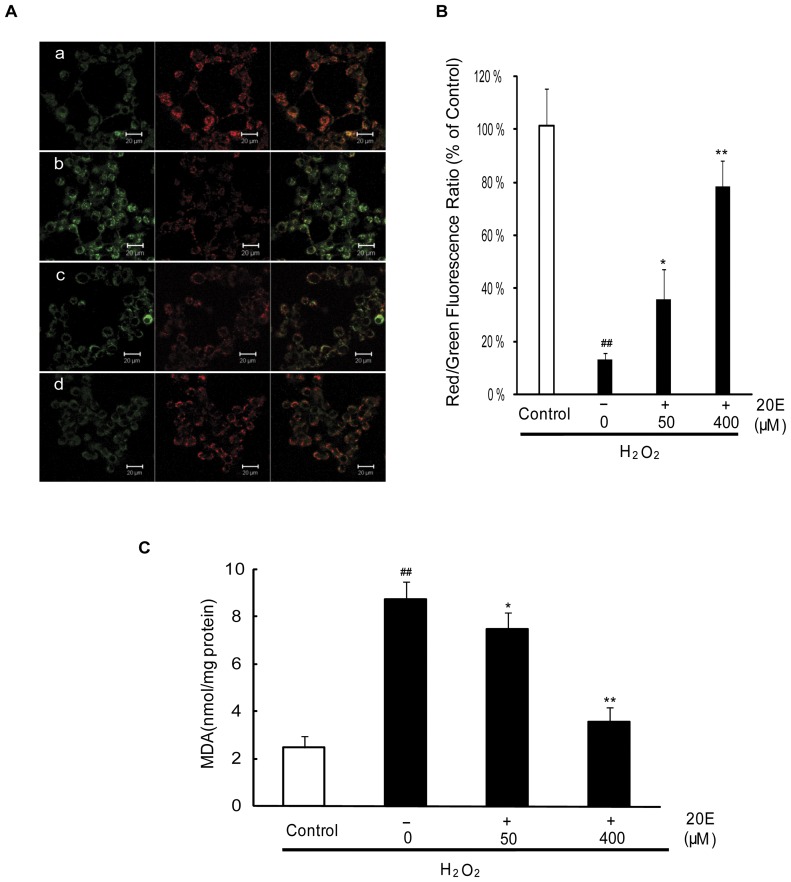
Effect of 20E on mitochondrial membrane potential and lipid peroxidation in H_2_O_2_-treated B35 cells. Cells were treated as Figure 3 and then incubated with the membrane potential indicator JC-1. A: Intracellular red and green fluorescence of JC-1 were determined by confocal laser microscopy. a: control; b: H_2_O_2_ treatment; c: 50 µM 20E-pretreatment for 24 h before H_2_O_2_ exposure; d: 400 µM 20E-pretreatment for 24 h before H_2_O_2_ exposure. B: The intracellular red and green fluorescence of JC-1 was measured by flow cytometry. Histogram showing the red/green fluorescence intensity ratio in different treatment cells. Data are reported as the means ± S.D. from three independent experiments. Non-treated cells served as controls. ^##^ *p*<0.01 vs. Control, ^*^ *p*<0.05 vs. H_2_O_2_ alone, ^**^ *p*<0.01 vs. H_2_O_2_ alone. C: Histogram showing MDA level in different treatment cells. Data are reported as the means ± S.D. from four independent experiments in triplicate. Non-treated cells served as controls. ^##^ *p*<0.01 vs. Control, ^*^ *p*<0.05 vs. H_2_O_2_ alone, ^**^ *p*<0.01 vs. H_2_O_2_ alone.

Mitochondria are an important cellular target of oxidative stress in neurons under ischemic conditions [7]. Oxidative stress leads to mitochondrial membrane damage and disruption of metabolism and redox status, which alters MMP dissipation. MMP dissipation is believed to be a common early event of apoptosis [35]. In this study, the lipophilic cationic probe JC-1 was used to evaluate mitochondrial transmembrane potential. As shown in Fig. 4A, B, the control cells were stained with JC-1, which clearly appeared red. However, exposure to 200 µM H_2_O_2_ for 12 h rapidly caused MMP dissipation, as shown by the increase in green fluorescence and the concomitant disappearance of red fluorescence, which decreased the red/green fluorescence intensity ratio (13.31±2.80% of control). Pretreatment with either 50 or 400 µM 20E for 24 h before H_2_O_2_ exposure attenuated the changes in MMP as indicated by repression of green fluorescence and restoration of red fluorescence. The red/green fluorescence intensity ratio was increased to 39.26±9.46% of control and 78.33±9.60% of control, respectively.

Lipid peroxidation product is widely used as a marker of cell membrane damage induced by oxidative stress [4]. In this study, MDA, the end product of lipid peroxidation was used to evaluate lipid peroxidation. Exposition to 200 µM H_2_O_2_ for 12 h caused an increase in the levels of intracellular MDA from 2.49±0.47 to 8.75±0.75 nmol/mg protein. However, pretreatment with either 50 or 400 µM 20E for 24 h before H_2_O_2_ exposure dramatically decreased the levels of intracellular MDA to 7.49±0.7 and 3.56±0.61 nmol/mg protein, respectively (Fig. 4C).

### 20E inhibits oxidative stress-induced [Ca^2+^]i elevation

**Figure 5:**
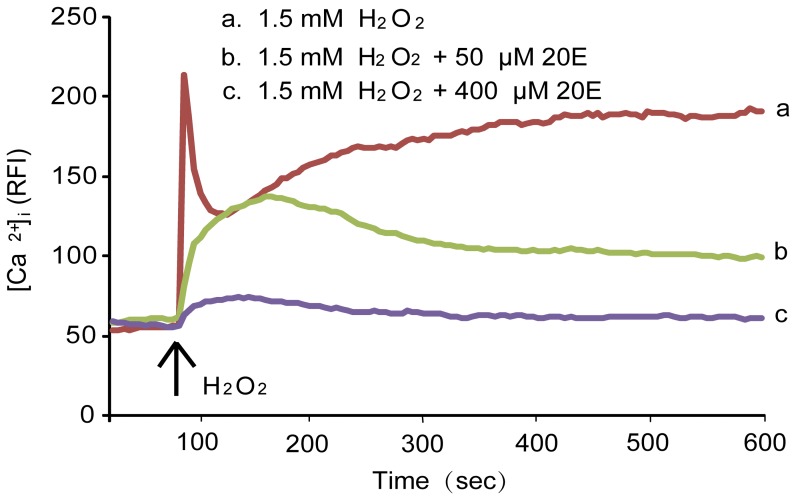
Effect of 20E on H_2_O_2_-induced elevation of [Ca^2+^]i in B35 cells. Cells were pretreated with 50 or 400 µM 20E for 1 h. These treated cells were labeled with 5 µM fluo-3-acetoxymethyl (AM) for 40 min and treated with 1.5 mM H_2_O_2_ for the indicated time. [Ca^2+^]i was monitored using a laser scanning confocal microscope as explained in the “Materials and Methods”. All images (120 images per cell) were processed to analyze changes in [Ca^2+^]i at the single cell level. The results are expressed as the relative fluorescence intensity (RFI). Each trace shows a single cell from three independent experiments.

An increase of [Ca^2+^]i is involved in oxidative stress-induced cell injury in many studies [11]. We used a Ca^2+^ imaging technique to observe the [Ca^2+^]i response induced by H_2_O_2_ in the presence or absence of 20E in B35 neural cells. As shown in Fig. 5, [Ca^2+^]i rapidly increased with treatment with 1.5 mM H_2_O_2_, but pretreatment with 50 or 400 µM 20E for 1 h showed concentration-dependent inhibition of the 1.5 mM H_2_O_2_-induced elevation of [Ca^2+^]i.

### 20E suppresses oxidative stress-induced NO production and iNOS expression by blocking NF-κB activation

**Figure 6:**
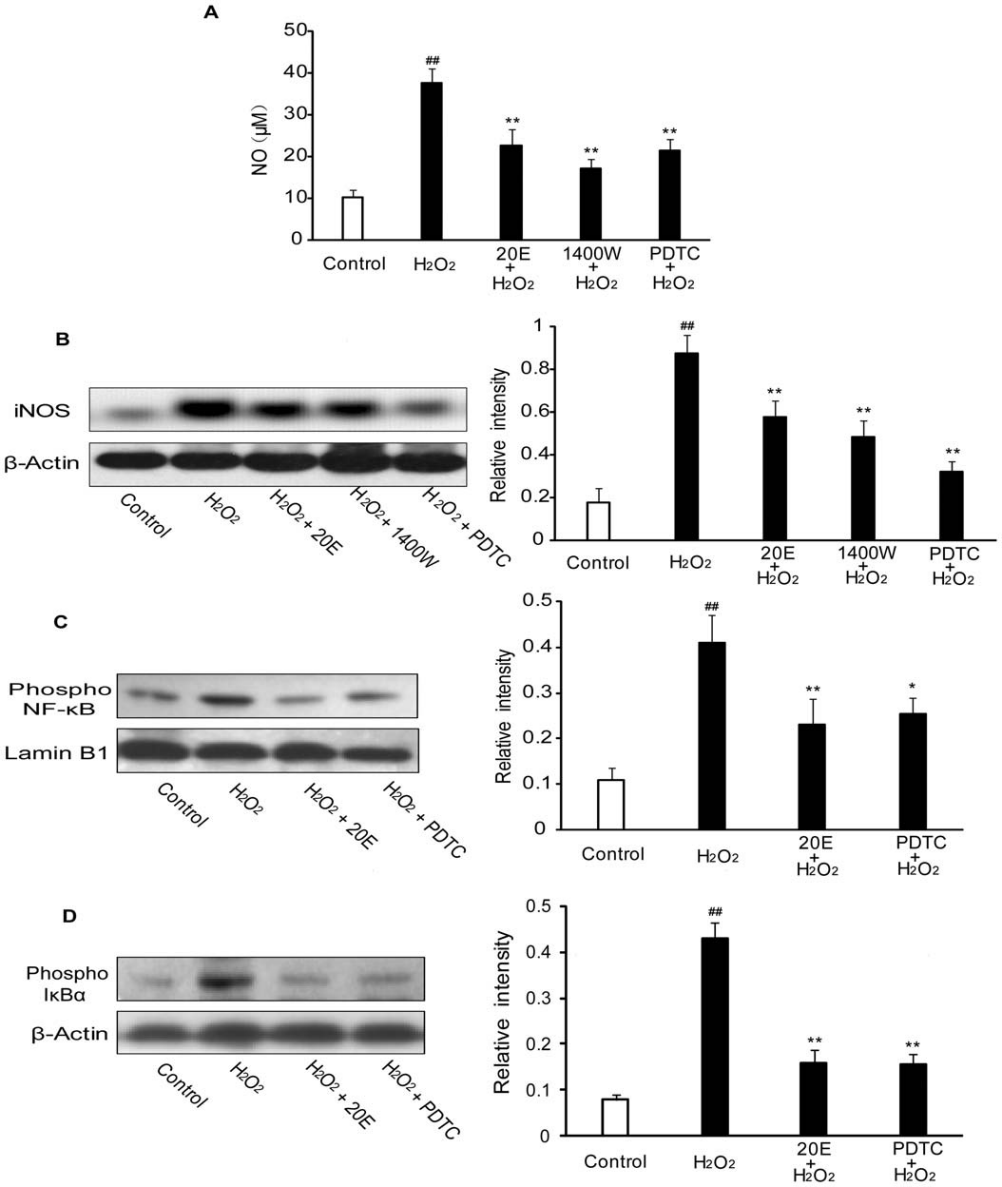
Effect of 20E on NO, iNOS and activation of NF-κB in H_2_O_2_-treated B35 cells. A: 20E inhibited H_2_O_2_-induced NO production. Cells were pretreated with 20E (400 µM) for 24 h or 1400W (200 µM) for 1 h or PDTC (100 µM) for 1 h before a 12 h incubation with H_2_O_2_. The production of nitrite in the medium was measured. Data are reported as the means ± S.D. from six experiments with 5×10^5^ cells/experiment. Non-treated cells served as controls. ^##^ *p*<0.01 vs. Control, ^**^ *p*<0.01 vs. H_2_O_2_ alone. B: 20E inhibited H_2_O_2_-induced iNOS expression. Cells were pretreated with 20E (400 µM) for 24 h or 1400W (200 µM) for 1 h or PDTC (100 µM) for 1 h before a 12 h incubation with H_2_O_2_. The iNOS expression was analyzed by western blot. The relative intensities of the bands were measured using quantity one software. C–D: 20E inhibited H_2_O_2_-induced NF-κB activation. Cells were pretreated with 20E (400 µM) for 24 h or PDTC (100 µM) for 1 h before a 12 h incubation with H_2_O_2_. The expression of phosphorylated IκBα (cytosolic part) and phosphorylated NF-κB p65 (in the nuclear extract) was analyzed by western blot. The relative intensities of the bands were measured using quantity one software. Data are reported as the means ± S.D. from three independent experiments. Non-treated cells served as controls. ^##^ *p*<0.01 vs. Control, ^*^ *p*<0.05 vs. H_2_O_2_ alone, ^**^ *p*<0.01 vs. H_2_O_2_ alone.

NO and iNOS have been implicated in oxidative stress apoptotic cell death [3]. In addition, production of NO and expression of iNOS are upregulated under ischemic conditions. Therefore, we examined the effect of 20E on H_2_O_2_-induced production of NO and expression of iNOS. The production of NO was evaluated by a colorimetric reaction with Griess reagent. As shown in Fig. 6A, exposure to 200 µM H_2_O_2_ for 12 h induced a rapid rise in the production of nitrite in the medium, from 10.33±1.76 to 37.59±3.51 µM. However, 20E and N-([3-(aminomethyl)phenyl]methyl) ethanimidamide dihydrochloride (1400W), a selective iNOS inhibitor, significantly decreased the nitrite content to 22.55±4.04 µM (*p*<0.01 in comparison to the H_2_O_2_ treatment group) and 17.1±2.22 µM (*p*<0.01 in comparison to the H_2_O_2_ treatment group), respectively (Fig. 6A). In addition, western blot revealed an increase in iNOS levels in the cytosol after B35 neural cell exposure to H_2_O_2_ alone. However, 20E attenuated the expression of the iNOS, as did 1400W (*p*<0.01 in comparison to the H_2_O_2_ treatment group) (Fig. 6B).

NF-κB activity was analyzed by western blot to explore the mechanisms underlying the inhibitory effect on iNOS protein expression by 20E after exposure to H_2_O_2_. The transcriptional activity of NF-κB can be regulated by the phosphorylation of IκB as well as the NF-κB p65 subunit. In our study, we observed that H_2_O_2_ elevated the expression of phosphorylated IκBα in the cytosol and phosphorylated NF-κB p65 in the nucleus. However, 20E was as effective in preventing the phosphorylation of IκBα and NF-κB p65 as pyrrolidine dithiocarbamate (PDTC), a potent NF-κB inhibitor (Fig. 6C, D). Further, PDTC directly decreased the expression of the iNOS and nitrite contents in the medium, as did 20E and 1400W (*p*<0.01 in comparison to the H_2_O_2_ treatment group) (Fig. 6A, B).

### 20E prevents the phosphorylation of ASK1, MKK4/7 and JNK induced by oxidative stress in B35 neural cells

**Figure 7:**
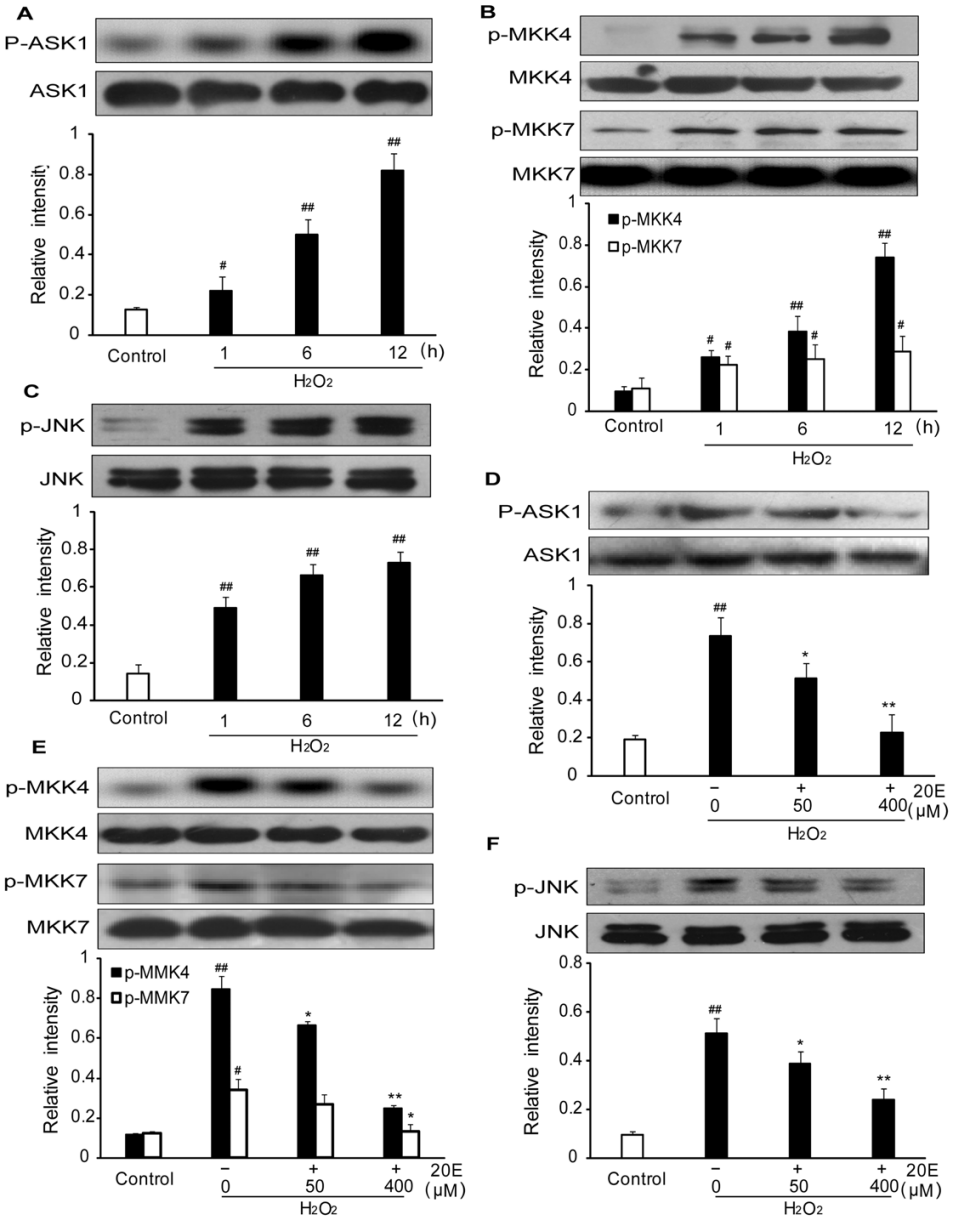
Effects of 20E on H_2_O_2_-induced phosphorylation of ASK1, MKK4/7 and JNK in B35 cells. A–C: H_2_O_2_ induced phosphorylation of ASK1, MKK4/7 and JNK. Cells were treated with 200 µM H_2_O_2_ for 1 h, 6 h or 12 h. Western blotting analysis of intracellular phosphorylation level of ASK1, MKK4/7 and JNK. The relative intensities of the bands were measured using quantity one software. D–F: 20E inhibited H_2_O_2_-induced phosphorylation of ASK1, MKK4/7 and JNK1/2. Cells were pretreated with 50 or 400 µM 20E for 24 h before a 12 h incubation with H_2_O_2_. Western blotting analysis of intracellular phosphorylation level of ASK1, MKK4/7 and JNK. The relative intensities of the bands were measured using quantity one software. Data are reported as the means ± S.D. from three independent experiments. Non-treated cells served as controls. ^#^ *p*<0.05 vs. Control, ^##^ *p*<0.01 vs. Control, ^*^ *p*<0.05 vs. H_2_O_2_ alone, ^**^ *p*<0.01 vs. H_2_O_2_ alone.

ROS/RNS are very unstable and highly reactive, and they tend to initiate chain reactions that result in irreversible chemical changes in proteins or lipids. In addition, ROS/RNS also directly initiate signal transduction pathways, which leads to injury [3]. ASK1-MKK4/7-JNK signaling pathway is involved in regulating oxidative stress-induced damage. We examined the phosphorylation levels of ASK1, MKK4/7 and JNK1/2 in B35 cells after exposition to H_2_O_2_ alone for different times or pretreatment with different concentrations of 20E before the onset of H_2_O_2_ treatment to investigate whether the ASK1-MKK4/7-JNK signaling pathway is involved in the neuroprotective mechanisms of 20E. Western blot analysis showed that at 1, 6 h and 12 h after exposure to H_2_O_2_, there was a significant increase in the phosphorylation of ASK1 compared with the control group. Pretreatment with either 50 or 400 µM 20E for 24 h before H_2_O_2_ exposure inhibited the phosphorylation of ASK1 induced by H_2_O_2_. Total ASK1 did not change in any group (Fig. 7A, D). The results were similar in the cases of MKK4/7 and JNK (Fig. 7B, C, E, and F).

### 20E protects rats against transient focal cerebral ischemic injury

**Figure 8:**
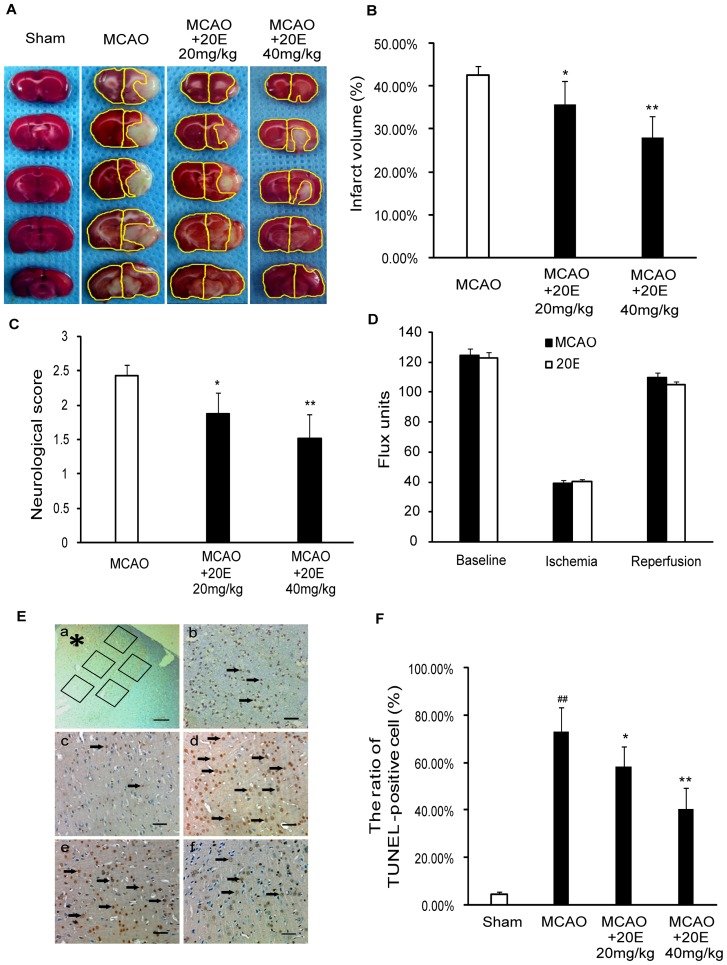
Protective effects of 20E on cerebral ischemia in rats. A: 20E attenuated transient MCAO-induced infarction assessed by TTC staining. After 24 h of reperfusion after 2 h of MCAO in rats, five consecutive TTC-stained coronal brain slices arranged in cranial to caudal order are shown. The white brain area represents infarcted tissue. B: Percentage of infarct areas in TTC-stained brain sections (n = 8 per group). C: 20E inhibited MCAO-induced neurological deficits in rats. After 24 h of MCAO reperfusion, the neurological status for the animals was evaluated (n = 8 per group). D: Changes in rCBF were not different between operated and 20E-treated groups (n = 4 per group) during or after MCAO in rats. E: Photographs of TUNEL staining in the ipsilateral cerebral cortex. (a) Five random and nonoverlapping regions were selected in the border of the ischemic cortical area. (b) Only densely labeled cells (arrows) were considered as positive apoptotic cells. (c) Sham-operated group; (d) MCAO group; (e) group treated with 20 mg/kg 20E; (f) group treated with 40 mg/kg 20E. TUNEL-positive cells are indicated by black arrows. *, ischemic cortical area; Scale bar, 200 µm in A; Scale bar, 50 µm in b–f. F: The percentage of TUNEL-positive cells described as the number of TUNEL-positive cells/total number of cells in each field (n = 6 per group). Data are reported as the means ± S.D. ^##^ *p*<0.01 vs. Sham-operated group, ^*^ *p*<0.05 vs. MCAO group, ^**^ *p*<0.01 vs. MCAO group.

To further assess whether 20E had neuroprotective effects against cerebral ischemia in vivo, a model of transient focal ischemia produced by MCAO was employed. MCAO for 2 h, followed by a 24-h reperfusion, resulted in a large ipsilateral cerebral infarction, which was shown by a white region after TTC staining at 24 h of reperfusion. An intraperitoneal injection of 20E (20 or 40 mg/kg in rats) at the onset of reperfusion significantly reduced the cerebral infarction area induced by MCAO (Fig. 8A). Fig. 8B summarizes the effects of 20E (20 and 40 mg/kg) on cerebral infarction in this rat brain focal ischemia model. 20E (20, 40 mg/kg) significantly decreased the total infarct volume to 34.62±5.48% (*p*<0.05 in comparison to the MCAO group) and 27.75±5.22% (*p*<0.01 in comparison to the MCAO group) of the coronal sections, respectively, from 42.53±2.14% of the coronal sections. Furthermore, 20E (20, 40 mg/kg) also reduced the neurological deficit scores to 1.88±0.31 (p<0.05 in comparison to the MCAO group) and 1.52±0.35 (p<0.01 in comparison to the MCAO group), respectively, compared to sham-operated rats that scored 2.43±0.15 (Fig. 8C).

**Figure 9:**
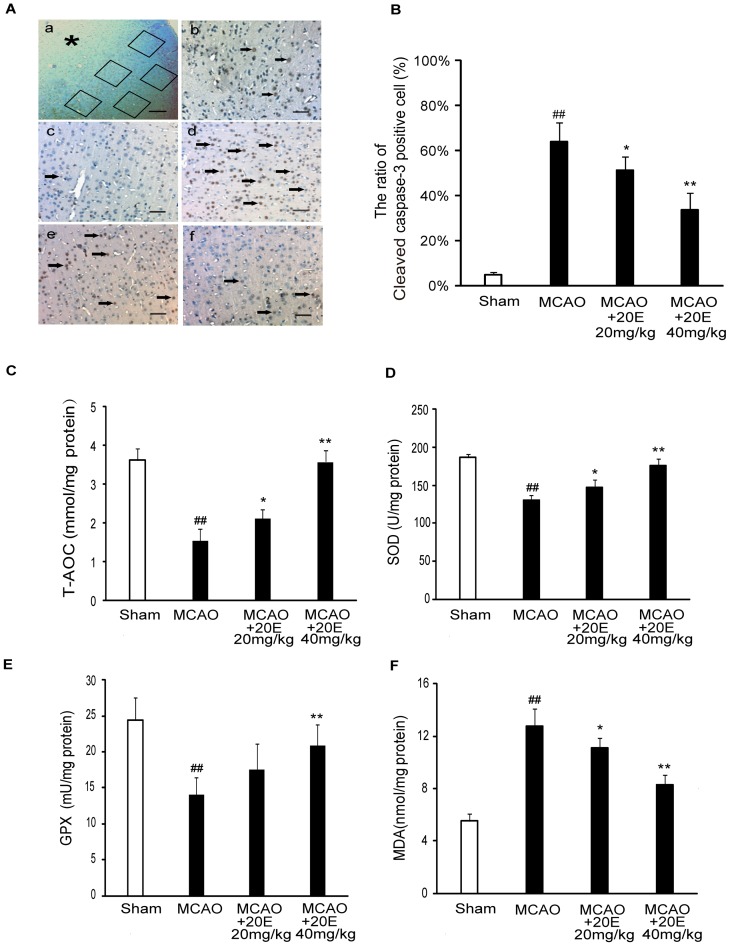
Effects of 20E on cleaved caspase-3, T-AOC,SOD,GPX and MDA in ischemic cortex in rat. A: Photographs of immunohistochemistry staining for cleaved caspase-3 in the ipsilateral cerebral cortex. (a) Five random and nonoverlapping regions were selected in the border of the ischemic cortical area. (b) Only densely labeled cells (arrows) were considered as positive apoptotic cells. (c) Sham-operated group; (d) MCAO group; (e) group treated with 20 mg/kg 20E; (f) group treated with 40 mg/kg 20E. Cleaved caspase-3-positive cells are indicated by black arrows. *, ischemic cortical area; Scale bar, 200 µm in A; Scale bar, 50 µm in b–f. B: The percentage of cleaved caspase-3-positive cells described as the number of cleaved caspase-3-positive cells/total number of cells in each field (n = 6 per group). C: Histogram showing cellular T-AOC in different treatment groups (n = 8 per group). D: Histogram showing cellular SOD activities in different treatment groups (n = 8 per group). E: Histogram showing cellular GPX levels in different treatment groups (n = 8 per group). F: Histogram showing MDA levels in different treatment groups (n = 8 per group). Data are reported as the means ± S.D. ^##^ *p*<0.01 vs. Sham-operated group, ^*^ *p*<0.05 vs. MCAO group, ^**^ *p*<0.01 vs. MCAO group.

Apoptotic cell death was determined by TUNEL staining and immunohistochemistry staining for cleaved caspase-3. As shown in Fig. 8E–F, the percentage of TUNEL-positive cells significantly increased in the infarct region in the MCAO group (73.15±10.37%), whereas TUNEL staining was negative (4.52±1.13%) in sham-operated rats. Treatment with 20E (20, 40 mg/kg) significantly reduced the percentage of TUNEL-positive cells in the ipsilateral cortex to 58.15±8.64% (*p*<0.05 in comparison to the MCAO group) and 40.37±9.15% (*p*<0.01 in comparison to the MCAO group), respectively, compared with the MCAO group. Consistent with the TUNEL analysis data, the percentage of cleaved caspase-3-positive cells increased from 4.83±1.17% to 64.00±8.70% in the infarct region in the MCAO group. However, the percentage of cleaved caspase-3-positive cells was significantly reduced to 51.14±6.04% (*p*<0.05 in comparison to the H_2_O_2_ treatment group) and 33.83±7.52% (*p*<0.01 in comparison to the H_2_O_2_ treatment group), respectively, when rats were treated by 20E (20, 40 mg/kg) (Fig. 9A, B). To rule out the possibility that 20E might have altered rCBF, and thereby protect against ischemic injury, we also monitored rCBF in both operated and 20E-treated rats. As shown in Figure 8D, no difference in rCBF changes was detected between the two groups before, during, or after MCAO. Altogether, these findings suggested that 20E reduced infarct volumes induced by MCAO in the rat brain, improved neurologic function and inhibited cell apoptosis after MCAO and provided neuroprotection against cerebral ischemia.

### 20E attenuates antioxidant potential descent and lipid peroxidation in the rat cortex induced by transient focal cerebral ischemia

To evaluate oxidative damage following cerebral ischemia and the anti-oxidative effects of 20E in rats, we measured T-AOC, SOD activity, GPX activity level and MDA level in the rat cortex. As shown in Fig. 9C–E, the T-AOC, SOD activity and GPX activity level in the rat ischemic cortex were reduced from 3.62±0.31 to 1.55±0.3 mmol/mg protein, 186.44±5.21 to 129.99±6.75 U/mg protein and 24.42±3.04 to 14.06±2.37 mU/mg protein, respectively. However, 20E (20, 40 mg/kg) dramatically increased the T-AOC, SOD activity and GPX activity level to 2.12±0.24 or 3.58±0.3 mmol/mg protein, 146.88±11.18 or 176.29±8.84 U/mg protein and 17.49±3.66 or 20.83±2.97 mU/mg protein, respectively. In addition, rat focal ischemia also caused an increase in the levels of MDA in ischemic cortex from 5.51±0.54 to 12.75±1.3 nmol/mg protein, which was dramatically decreased by 20E (20, 40 mg/kg) to 11.09±0.71 and 8.3±0.72 nmol/mg protein, respectively (Fig. 9F).

## Discussion

Oxidative stress is a major cause of neuronal injuries induced by cerebral ischemia [3], [6]. H_2_O_2_ has been extensively used as an inducer of oxidative stress [36]. Thus, we used H_2_O_2_-induced injury in B35 neuroblastoma cells in vitro to simulate the oxidative damage following cerebral ischemia, attempting to search for a naturally occurring drug with neuroprotective effects. 20E, an insect steroid hormone, exhibits a potent antioxidative effect. This study demonstrates that 20E reversed the H_2_O_2_-induced cell injury, which was characterized by a decrease in cell viability, release of LDH, and mitochondrial membrane potential dissipation and increases in the number of TUNEL-positive and cleaved caspase-3-positive cells. Four specific actions by 20E may contribute to this neuroprotective effect: first, 20E inhibits the generation of ROS and restores cellular antioxidant potential; second, 20E blocks oxidative stress-induced increases of [Ca^2+^]i; third, 20E reduces the production of NO and the expression of iNOS protein, which may be mediated by inhibition of NF-κB activation; fourth, 20E suppresses the activation of the ASK1-MKK4/7-JNK stress signaling pathway induced by oxidative stress. Finally, we establish a rat MCAO model to further confirm the neuroprotective effect of 20E. The results of our in vivo studies showed that 20E significantly attenuated ischemia injury caused by MCAO in rats. These findings indicate that 20E is useful as a potential protective agent against cerebral ischemic injury.

Ischemia could increase production of ROS and undermine antioxidant defense in brain tissues, which causes an imbalance of pro-oxidants and antioxidants [4]. Furthermore, excessive production of ROS may lead to cell injury by damaging cellular macromolecules [5], [8]. However, these effects can be inhibited or delayed by a wide variety of antioxidants [37], [38]. 20E has anti-radical properties and efficiently quenchs ROS because it can react with radicals by abstracting hydrogen from carbon 9 at a B–C ring junction and [26]. Many studies have shown that 20E can inhibit lipid peroxidation and stabilize mitochondrial membrane potential [25], [30]. In accordance with this notion, we observed that 20E could significantly inhibit H_2_O_2_-induced intracellular ROS production, as revealed by reduced distribution of the DCF-DA fluorescent dye in cells pretreated with 20E. On the other hand, we also observed that H_2_O_2_ decreased intracellular T-AOC, SOD activity and GPX activity level and that treatment with 20E markedly attenuated the above changes, which suggested that 20E enhanced cellular antioxidant defense activities. Accumulating evidence demonstrates that mitochondria are an important cellular target of oxidative stress [39]. Oxidative stress depolarizes mitochondrial membrane potential by lipid peroxidation, which further leads to mitochondrial dysfunction. Mitochondria play a critical role in apoptosis signaling pathways, which are associated with cerebral ischemia injury [40]. Mitochondrial dysfunction could result in cell death by release of mitochondrial proteins into the cytosol and subsequent activation of cell death execution molecules [41], [42]. In this study, H_2_O_2_ could cause the increases of MDA, the end product of lipid peroxidation, and depolarization of mitochondrial membrane potential in cells, both of which were alleviated significantly by pretreatment with 20E. Together, these results support that 20E could reduce ROS accumulation, protect antioxidant enzymes, prevent lipid peroxidation and preserve the structural and functional integrity of mitochondria in H_2_O_2_-treated B35 neural cells.

There is increasing evidence that cerebral ischemia leads to an increase in [Ca^2+^]i [43]. Ischemia restricts the delivery of metabolic substrates, particularly oxygen and glucose, leading to depletion of the energy stores required to maintain ionic gradients. With energy depletion, membrane potential is lost and neurons depolarize, which finally leads to increased [Ca^2+^]i. Moreover, the elevation in [Ca^2+^]i causes ROS generation by activating enzymes. Conversely, ROS generation can facilitate [Ca^2+^]i increases by damaging the [Ca^2+^]i regulatory mechanism and activating Ca^2+^ release from intracellular Ca^2+^ stores [44], [45]. Finally, the above effects cause intracellular calcium overload, which sets into motion a cascade of pathobiochemical processes that lead to neuronal degeneration [43], [44]. Previous study had proved that 20E inhibited the increase of [Ca^2+^]i in the skeletal muscles of rats suffered from D-hypovitaminosis [23]. In this study, we also observed that 20E markedly inhibited H_2_O_2_-induced [Ca^2+^]i elevation, suggesting that 20E can also attenuate H_2_O_2_-induced B35 neural cell injury by preventing intracellular calcium overload.

NO is an important mediator of oxidative damage after cerebral ischemia. However, the action of NO is a double-edged sword. NO is beneficial as a messenger or modulator, but it is potentially toxic under conditions of oxidative stress. The toxic effects of NO may be attributed to its free radical character, which makes NO react with certain proteins containing heme-iron prosthetic groups, iron-sulfur clusters, or reactive thiols. In addition, NO also reacts readily with superoxide (O_2_^−^) to produce a strong oxidant, peroxynitrite (ONOO^−^), which may mediate much of the NO toxicity [14]. Many studies have demonstrated that ischemia causes brain damage by excessive production of NO through the induction of iNOS. Neuropathophysiological symptoms in ischemic animal models were prevented by aminoguanidine and 1400 W, both selective iNOS inhibitors [46]. Furthermore, mice with a null mutation of the iNOS gene had reduced susceptibility to focal cerebral ischemia [47], [48]. These findings strongly suggest that the production of NO and the expression of iNOS protein are involved in the mechanisms of cerebral ischemic injury. In this study, we observed that H_2_O_2_ induced increases in the production of NO and the expression of iNOS protein were restored by 20E to a similar degree as 1400W, a selective iNOS inhibitor. These data suggest that 20E protected B35 neural cells against H_2_O_2_-induced injury by reducing iNOS expression and NO production.

Oxidative stress might contribute to NF-κB activation in cerebral ischemia [15]. Overexpression of ROS-scavenging enzymes, or superoxide dismutases (SOD) 1, protected against ischemic cell death in transgenic mice. In mice overexpressing SOD1, the activation of NF-κB in the ischemic brain is suppressed when compared with wild-type mice [49]. Moreover, cumulative evidence suggests that NF-κB is activated in cerebral ischemia, mainly in neurons, and contributes to neuronal cell death [50], [51]. NF-κB is composed of a homodimer or heterodimer of Rel protein. Among the NF-κB subunits, p65 and p50 are responsible for the detrimental effect in cerebral ischemia [52]. Some antioxidants and antioxidant enzymes inhibit the activation of NF-κB and reduce neuronal damage after cerebral ischemia [11]. In addition, Feng et al. observed that 20E inhabited the activation of NF-kappaB induced by H_2_O_2_ in the human lens epithelial cells [53]. So we investigated whether inhibition of H_2_O_2_-induced NF-κB activation was involved in the neuroprotection afforded by 20E. In most cases, NF-κB is kept inactive in the cytoplasm through binding of its inhibitor IκB. NF-κB activation in response to various extracellular signals requires IκB kinase activation, which phosphorylates IκBα, leading to IkBα's degradation. Free NF-κB dimers are then rapidly translocated to the nucleus, wherein they bind to the DNA and activate transcription of target genes. At the same time, phosphorylation at multiple serine sites of the p65 subunit increases the transcriptional activity of NF-κB in the nucleus. In this study, we observed that phosphorylation of the p65 subunit in the nucleus under oxidative stress conditions is linked with the simultaneous increased phosphorylation of IκBα in the cytosol, both of which were inhibited by 20E to a similar degree as PDTC, a potent NF-κB inhibitor. These data support that 20E depressed H_2_O_2_-induced NF-κB activation in B35 neural cells. Further, PDTC and 20E also reduced iNOS expression and NO production induced by H_2_O_2_ in B35 neural cells. These results are consistent with several studies that have shown that NF-κB activation is a key factor in the production of iNOS and NO. These data suggest that 20E reduced iNOS expression and NO production by suppressing activated NF-κB in H_2_O_2_ -treated B35 neural cells.

Besides directly damaging effects, ROS/RNS have been found to initiate apoptosis signaling pathways [11], [12], [54]. A growing body of evidence has suggested that ROS/RNS are potent inducers of c-Jun N-terminal kinases (JNK) activation [10]. JNK, also known as stress-activated protein kinase, is an important member of the mitogen-activated protein kinases (MAPK) superfamily. The specific molecular targets of JNK are closely related to apoptotic cell death factors [10]. The ischemic/hypoxic condition is accompanied by the production of ROS/RNS. Thus, cerebral ischemia causes JNK activation. Inhibition of JNK activation would significantly reduce apoptosis induced by cerebral ischemia [11], [12]. In addition, activation of JNK has been observed in many neuronal ischemic models, and SP600125, a selective inhibitor of JNK, provides robust and long-term neuroprotection and improved neurological function after ischemia [55]. In the present study, we observed that H_2_O_2_ induced the activation of JNK in B35 neural cells, which was inhibited by 20E. These data suggest that inhibition of JNK activation induced by oxidative stress is involved in the neuroprotective mechanisms of 20E.

Because 20E-mediated neuroprotection appears to depend on the inhibition of JNK activation, we further investigated components of the JNK kinase signaling pathway targeted by 20E. Recently, ASK1 was found to be an upstream kinase of JNK [10]. Various ASK1-dependent pathways are involved in JNK activation induced by multiple cell stressors such as reactive oxygen species and DNA damage [56], [57]. For example, ROS from either exogenous or endogenous sources induces ASK1 activation, which further activates both JNK and p38 by activating respective MAPKKs (JNKK1/MKK4, JNKK2/MKK7, MKK3, and MKK6) [56], [58]. ASK1 may be an important converging point of multiple pro-death signals that participate in ischemic neuronal death. Knockdown of ASK1 attenuates ischemia-induced JNK and p38 activation and cerebral ischemia insult. Thus, targeting ASK1 provides not only neuroprotection, but also more comprehensive protection of brain tissue [59]. It has been reported that the ASK1-MKK4-JNK pathway is an important pro-apoptosis signaling pathway following cerebral ischemia [12], [60]. This report led us to hypothesize that 20E can inhibit JNK activation via the ASK1-MKK4/7-JNK pathway to prevent the neuronal death induced by H_2_O_2_. In this study, we observed that pretreatment with 20E inhibited the activations of ASK1 and MKK4/7, which are linked with the simultaneous suppression of JNK activation by 20E in B35 neural cells under H_2_O_2_-treatment. These data support that 20E prevents H_2_O_2_-induced neuronal death through inhibition of the ASK1-MKK4/7-JNK pathway.

To confirm the neuroprotective effect of 20E, we investigated whether 20E could prevent ischemia-induced brain damage in an animal model. The rat MCAO model with reperfusion partly mimics the features of stroke in humans because the middle cerebral artery, which is the specific occlusion site in this model, is the most commonly affected vessel in both embolic and thrombotic strokes in humans [61], [62]. Therefore, we used a rat MCAO model with reperfusion to study the neuroprotective effects of 20E. The present study demonstrated that 20E effectively decreased infarct volume and the number of TUNEL-positive and cleaved caspase-3-positive cells in the brain in the rat MCAO model with reperfusion. This result was further supported by an alleviation of the MCAO-induced neurological deficits when rats were administered 20E. Meanwhile, we also observed a significant increase in the levels of MDA and significant decreases in T-AOC, SOD activity and GPX activity level in brains 24 h after ischemic insult, which supported the hypothesis that MCAO caused oxidative stress in the brain. However, 20E could significantly attenuate oxidative stress in the brain by inhibiting the above changes. Our results indicate that 20E also had potent neuroprotective effects against ischemia-induced brain damage in vivo.

**Figure 10:**
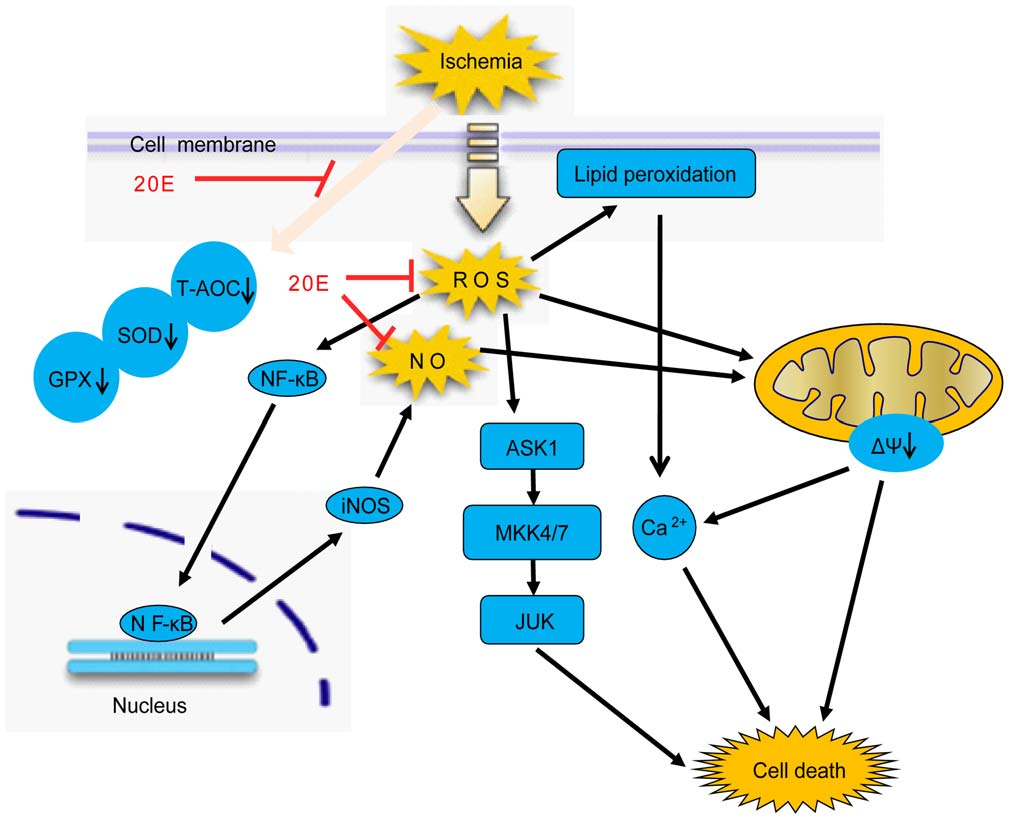
Multifunctional cytoprotective pathways are involved in the neuroprotection by 20E. Ischemia induces intracellular ROS accumulation, antioxidant potential descent and lipid peroxidation, which causes Intracellular oxidative stress. Further, oxidative stress leads to elevation of intracellular [Ca^2+^]i, the increases in NO production by NF-κB/iNOS pathway and the activation of ASK1-MKK4/7 -JNK stress signaling pathway in cells. Finally, the above changes cause cell death. However, 20E markedly attenuates the above changes and protect from cell death.

In summary, this study demonstrates that 20E is effective in reducing oxidative stress-induced cytotoxicity in B35 neural cells by inhibiting the generation of ROS and mitochondrial membrane potential dissipation, restoring cellular antioxidant potential, blocking the increases of [Ca^2+^]i, reduceing the production of NO and the expression of iNOS protein and suppressing the activation of the ASK1-MKK4/7-JNK stress signaling pathway, as shown in Fig. 10. Further, 20E showed alleviation of the MCAO-induced brain damage in a rat model. These features of 20E suggest that 20E is a promising therapeutic agent that potentially can be used to treat cerebral ischemic damage.
